# Biology of Cancer-Testis Antigens and Their Therapeutic Implications in Cancer

**DOI:** 10.3390/cells12060926

**Published:** 2023-03-17

**Authors:** Dawn Sijin Nin, Lih-Wen Deng

**Affiliations:** 1 Department of Biochemistry, Yong Loo Lin School of Medicine, National University of Singapore, MD 7, 8 Medical Drive, Singapore 117596, Singapore; bchdlw@nus.edu.sg; 2 NUS Center for Cancer Research, Yong Loo Lin School of Medicine, National University of Singapore, 14 Medical Drive, Singapore 117599, Singapore; 3 National University Cancer Institute, National University Health System, 5 Lower Kent Ridge Road, Singapore 119074, Singapore

**Keywords:** cancer-testis antigens, CT antigens, cancer

## Abstract

Tumour-specific antigens have been an area of interest in cancer therapy since their discovery in the middle of the 20th century. In the era of immune-based cancer therapeutics, redirecting our immune cells to target these tumour-specific antigens has become even more relevant. Cancer-testis antigens (CTAs) are a class of antigens with an expression specific to the testis and cancer cells. CTAs have also been demonstrated to be expressed in a wide variety of cancers. Due to their frequency and specificity of expression in a multitude of cancers, CTAs have been particularly attractive as cancer-specific therapeutic targets. There is now a rapid expansion of CTAs being identified and many studies have been conducted to correlate CTA expression with cancer and therapy-resistant phenotypes. Furthermore, there is an increasing number of clinical trials involving using some of these CTAs as molecular targets in pharmacological and immune-targeted therapeutics for various cancers. This review will summarise the current knowledge of the biology of known CTAs in tumorigenesis and the regulation of *CTA* genes. CTAs as molecular targets and the therapeutic implications of these CTA-targeted anticancer strategies will also be discussed.

## 1. Introduction

**Figure 1:**
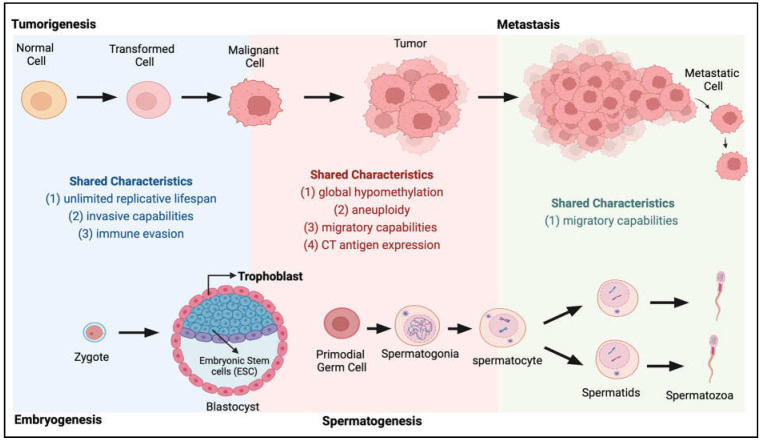
Shared characteristics between tumorigenesis and embryogenesis and spermatogenesis. During the transition from normal to tumour and metastasis (top panel), cancer cells acquire many characteristics highly similar to those present during normal development (bottom panel) when the zygote develops into an embryo and during the spermatogenesis process.

At the beginning of the 20th century, Scottish embryologist John Bread first proposed that cancer arises from migrating germ cells that were “displaced” and never reached the gonads during embryogenesis, a theory now known as the trophoblastic theory of cancer [1]. Many parallels at the molecular level can be drawn between the processes of embryogenesis, spermatogenesis and cancer progression (Figure 1), supporting this trophoblastic model for tumorigenesis. In the decades following Bread’s proposed theory, data suggesting frequent production of chorionic gonadotropin and other trophoblastic hormones in human cancers [2,3] further indicates the association between germ cell development and cancer progression. These discoveries galvanised the identification of a growing number of proteins that are exclusively expressed in trophoblasts, germ cells and tumour cells, proteins which we now know as cancer-testis antigens (CTAs) [4,5]. There is now a theory that the reactivation of the normally silenced gametogenesis programme in somatic cells could be a driving force of tumorigenesis [6] and these CTA proteins may play a role in signalling the pathways required for the acquisition of various biological capabilities leading to the cancer phenotype (acquisition of the Hallmarks of Cancer [7,8,9]).

CTAs were first identified through autologous genotyping [10,11]. With the advent of high-throughput PCR and sequencing techniques, there has been a rapid expansion of the number of CTA members identified [11,12,13,14,15,16,17], with more than 200 *CTA* genes curated in public databases [18]. However, how these genes are reactivated during tumorigenesis and how they contribute to cancer progression and therapy resistance remain to be studied. Although the role of CTAs in normal development and gametogenesis is also poorly understood, this review will focus on the role of CTAs in cancers, given the current interest in using CTAs as targets for immunotherapy. This review summarises the current knowledge on the biology of known CTAs in tumourigenesis and the regulation of *CTA* genes. CTAs as molecular targets and the therapeutic implications of these CTA-targeted anticancer strategies will also be discussed.

## 2. Discovery of CTAs

The earliest studies describing the presence of tumour antigens appeared in the 1950s [12,13,14,15] with observations that the murine immune system can clear carcinogen-induced tumours in murine models. These studies led to the search for human tumour-specific antigens, which can be used to redirect the immune system to cancer cells [16,17]. Lloyd J. Old and his group established the first human cancer antigens in the 1970s using a pioneering technique now known as autologous typing [10,11,18,19,20,21]. In 1991, using a combination of autologous typing and DNA cloning, van der Bruggen and colleagues identified a novel gene that directed the expression of antigen MZ2-E on a human melanoma cell line. As this gene was first identified as a melanoma antigen, the gene was coined *MAGE1* (melanoma-associated antigen 1), with the two other later identified family members from the same cell line named *MAGE2* and *MAGE3* [22]. The discovery of this family of *MAGE* genes was followed by the subsequent identification of the B melanoma antigen (*BAGE*) [23] and G antigen (*GAGE*) [24] family of genes using the same cell line. In-depth studies into the *MAGEA1* gene locus demonstrated that the *MAGEA* family consists of 12 closely related genes clustered at chromosome band Xq28, of which six are expressed in tumours [25,26,27]. The *MAGEB* cluster located on Xp21.3 was later identified while searching for the gene responsible for the sex-reversal phenotype [28]. A subsequent third cluster, the *MAGEC* genes, was identified in Xq26-27 [29]. More *MAGE* gene members have been progressively added to these two clusters [30,31] and other new genes, such as *SAGE* and *HAGE,* were also elucidated [32].

In the mid-1990s, a technological breakthrough in SEREX (serological analysis of cDNA expression libraries) further fuelled the expansion of CTA discovery [33]. SEREX utilises a recombinant cDNA phage expression library system with immuno-screening performed using autologous patient sera for tumour antigen identification. This approach circumvented several limitations of autologous typing, including establishing cytotoxic T-cell lines from tumour patients and establishing tumour cell lines from the autologous tumour specimens (both of which can be challenging) [34]. Unlike autologous typing, which detects mainly antigens presented on the cell surface, the advantage of SEREX is its ability to identify intracellular antigens, which increases the number of antigens that can be identified with this technology [35]. One of the most prominent CTAs identified using this technology is the highly immunogenic New York oesophageal squamous cell carcinoma 1 (NY-ESO-1), one of the most commonly used targets in CTA-targeted therapeutic strategies today. The term “cancer-testis antigen” was also first used to coin this unique family of proteins found to be expressed exclusively in testis and cancer cells in the seminal paper by Chen et al. describing the identification of NY-ESO-1 [35]. Further work subsequently demonstrated that CTAs are not only restricted to germ cells within the testis [36] but also in the immature germ cells of the female ovary [37] and trophoblasts. Therefore, cancer-germline antigens (CGAs) may be a more appropriate name for this class of proteins.

CTAs are broadly classified into two categories: those encoded on the X chromosome, known as CT-X antigens, and non-X CTAs, antigens with testis-restricted expression but encoded by non-X chromosomes (including those encoded by autosomes and genes such as *VCY* and *VCY1B* that are encoded by the Y chromosome [38,39]). Current candidates for cancer vaccines are mainly focused on CT-X antigens. Genes encoding CT-X antigens are typically expressed in spermatogonia and consist of several adjacent duplications of small genomic regions with > 99% similarities between copies (ampliconic genes [40]). Interestingly, only 31% of human X ampliconic genes have an ortholog in mice, suggesting a quick evolutionary turnover for these genes [41]. Approximately 10% of the genes on the X chromosome belong to CT-X families [42] and more than half of the now-identified *CTA* genes belong to the CT-X family [43]. In contrast, autosomal non-X *CTA* genes have a genome-wide distribution and do not generally form gene families or reside in genomic repeats. Non-X CTAs are also expressed predominantly in the later stages of spermatogenesis, such as in spermatocytes [44,45,46,47].

## 3. CTA Biology in Carcinogenesis

**Figure 2:**
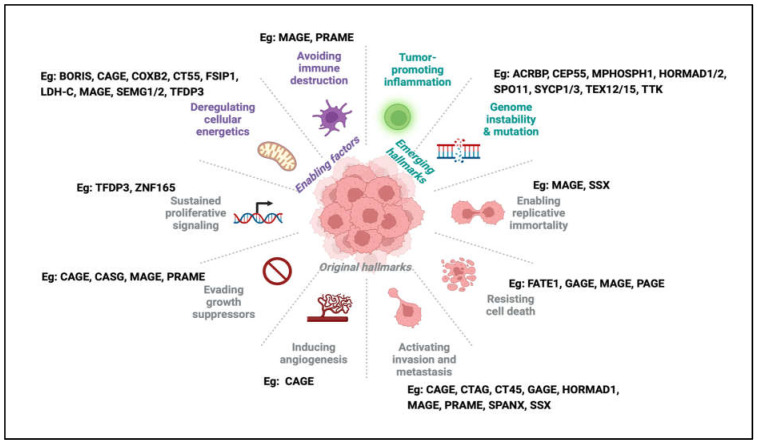
The hallmarks of cancer and examples of CTAs associated with each hallmark. In the latest update of the hallmarks of cancer, one can find examples of the involvement of CTAs in almost every hallmark, from original to newly emerging hallmarks.

Using the *Drosophila melanogaster* model, Janic et al. showed that Drosophila *CTA* genes could drive tumour growth and their suppression was sufficient to curb tumour growth [48]. To date, several studies have implicated individual CTAs in driving a multitude of cellular pathways leading to the cancer phenotype in human cells, suggesting the diversity of CTA function in human cancers [49] and their involvement in conferring or reactivating critical hallmarks of cancer (Figure 2).

### 3.1. CTAs in the Regulation of Transcriptional Programs Involved in Tumorigenesis

One of the earliest CTAs for which molecular function was defined was PRAME (preferentially expressed antigen in melanoma-CT130). As its name suggests, PRAME was first identified in melanoma and was shown to be causally involved in the tumourigenic process in melanoma [50]. Subsequently, PRAME was found to be expressed in several other cancers [51,52,53,54] and high PRAME expression was demonstrated to be an independent prognostic marker for poor outcomes in breast cancer and neuroblastoma [52,53]. PRAME was identified as a dominant repressor of retinoic acid receptor (RAR) signalling. Through its binding with RAR in the presence of retinoic acid (RA), PRAME prevented the ligand-induced transcriptional activation of RAR target genes by recruiting Polycomb proteins in the repressor complex. This PRAME–RAR interaction inhibits transcriptional programmes involved in RA-induced differentiation, growth arrest and apoptosis, which results in the cancer phenotype [55].

MAGE is probably one of the most prominent families of CTAs, and of the now-identified 60 proteins, 24 have been reported to be aberrantly expressed in various cancers [56]. MAGE-family proteins are characterised by the MAGE homology domain, an approximately 200 amino-acid domain usually located near the C-terminus. However, it can be more centrally located in some proteins [27]. Laduron et al. first reported the interaction between MAGEA1 and the Ski-interacting protein (SKIP) using a yeast-two hybrid assay. MAGEA1–SKIP interaction actively represses transcription activation by binding and recruiting histone deacetylase 1 (HDAC1) to SKIP target genes [57]. Given that most tumour cells have altered HDAC activities, it is reasonable to postulate that MAGEA1–SKIP-HDAC1 interaction may contribute to transcriptional alterations that favour tumour cell growth by recruiting HDAC1 to these SKIP target genes. Another MAGE family member, MAGEA2, was reported to affect the transactivation properties of potent tumour suppressor p53 by forming a potent p53 inhibitory loop involving histone deacetylase 3 (HDAC3) recruitment [58]. Marcar and colleagues later showed that MAGEA–p53 association occluded p53-DNA binding, resulting in the inability of p53 to bind to target promoters for transcription activation. The loss of p53-mediated activation of genes such as *p21*, *MDM2* and *PUMA* results in tumour progression [59].

In another study conducted using yeast-two hybrid screens, MAGE11 was found to interact with the FXXLF α-helical region of the androgen receptor (AR) [60]. Here, the MAGE11–AR interaction stabilised ligand-free AR expression, which could account for the increased levels of AR frequently observed with recurrent prostate cancer [61,62,63]. Further, MAGE11–AR interaction increased AR activity in the presence of agonists by increasing the exposure of the AR ligand-binding activation function 2 (AF2) site to the recruitment and activation by the SRC/p160 coactivators [60].

Apart from PRAME and MAGE, CAGE (cancer-associated antigen) was also reported to have effects on transcription by inducing the interaction of Snail with HDAC2, which negatively regulates p53 expression [64]. Recent work by Yang and Potts [65] likewise demonstrated that the CTA chondrosarcoma-associated gene 2 (CASG2) exerts multiple effects on transcription via its inhibitory binding to the NAD+-dependent deacetylase SIRT1. CASG2–SIRT1 binding inhibits SIRT1 deacetylase activity, impacting downstream events ranging from chromatin remodelling to p53- and other SIRT-mediated gene regulatory functions. The CTA transcription factor DP family member 3 (TFDP3) was also shown to be an E2F partner and together modulates the expression of E2F target genes involved in cell proliferation and apoptosis, not only in cancer but in the testis as well [66].

Transcriptional activity modulated by transforming growth factor beta (TGFβ) signalling is highly context-dependent with oncogenic and tumour-suppressive roles. While TGFβ seems more tumour-suppressive in luminal A/B subtypes in breast cancer, it has an oncogenic role in triple-negative breast cancer (TNBC) [67,68]. Recent data from a loss-of-function screen for the transformation and proliferative properties of TGFβ signalling in TNBC revealed the function of the CTA ZNF165 (a C_2_H_2_ zinc finger transcription factor) in the oncogenic properties of TGFβ. ZNF165 was found to be associated with several TGFβ loci activating or repressing transcription, tipping the balance to a more oncogenic transcriptional programme [69]. In a following study, it was further shown that ZNF165 association with SMAD3 modulates the transcription of TGFβ-dependent genes. It was further demonstrated that the KRAB zinc finger protein ZNF446 and its associated tripartite motif protein TRIM27 are essential components of the ZNF165–SMAD3 transcription complex, highlighting the ability of CTAs to co-opt somatic transcriptional machinery to drive oncogenic pathways [70].

Closely related to the theme of transcriptional regulation, recent studies have also highlighted the potential role of CTAs in regulating chromatin organisation [71,72,73,74]. The ability of CTAs to affect chromatin structure further implicates their role in the gene regulatory network. One such example would be BORIS (the brother of the regulator of imprinted sites, also known as CTCFL). An elegant study by Debruyne and colleagues demonstrated that *ALK*-mutated, *MYCN*-amplified neuroblastoma cells that develop resistance to ALK inhibition had elevated BORIS expression and altered chromatin looping interactions. Changes in chromatin looping interactions resulted in the formation of super-enhancers that drove the ectopic expression of a subset of proneural transcription factors that ultimately defined the resistance phenotype in neuroblastoma cells [71]. Another CTA, the ATPase family AAA domain-containing protein 2 (ATAD2), was reported to be a reader of di-acetylated histone H4 at lysine 5 and lysine 12, two histone marks that mark newly synthesised histones during replication-coupled chromatin reassembly [72]. In embryonic stem cells, ATAD2 binding to nucleosomes guided by histone acetylation at active gene sites alters chromatin dynamics required for the proper activity of highly expressed gene fractions of the genome [74].

The emerging theme suggested by these studies is that CTAs can be used in transcriptional networks to alter or regulate gene regulatory programmes involved in cancer progression. There is also cumulating evidence involving other CTAs in transcriptional regulation, such as SSX in transcriptional repression in synovial sarcoma [75]. In addition, other CTA family members, such as GAGE, SPANX (sperm-protein-associated with the nucleus mapped to the X chromosome) and PAGE (prostate-associated gene), have been reported to be localised in the nucleus or nuclear envelope [73,76,77,78,79]. It is reasonable to postulate that the nuclear localisation of these CTAs may indicate their potential role in chromatin organisation or transcriptional regulation, which remains to be thoroughly explored. More in-depth mechanistic studies into how CTAs exert transcriptional control will not only improve our understanding of these proteins in cancer progression but also shed light on their possible functions in development and gametogenesis.

### 3.2. CTAs in Cell Division, Genomic Instability and the DNA Damage Response

Cancer cells are rapidly dividing and proper division of these cells is essential for their survival and continued propagation. Many CTAs have been implicated in the survival advantage of cancer cells via their functions in preventing catastrophic mitotic or genomic events in these rapidly dividing cells [80,81,82,83]. Of note, there is now increased interest in developing small-molecule inhibitors that target these CTAs to be used in conjunction with current chemotherapeutic mitotic poisons to reduce the cytotoxicity of these drugs [84]. The non-X CTA TTK (cancer-testis antigen 96 or CT96) was first identified by screening the T-cell expression library with antiphosphotyrosine antibodies [85]. TTK was observed to preferentially express in the testis and thymus, where cells were rapidly proliferating, but not in other tissues. Later studies on the homolog of TTK, Mps1, in *Xenopus* demonstrated that Mps1 is required to maintain mitotic checkpoint integrity via the recruitment and active retention of CENP-E at kinetochores, allowing proper progression into anaphase and protection against aneuploidy [86,87]. Elevated TTK expression was later described in several other cancers, such as breast [88], pancreatic [89], liver [90] and lung cancers [91], where TTK inhibition induced polyploidy and apoptosis in lung cancer cells, highlighting the therapeutic potential of TTK inhibitors in cancer treatment. To this end, multiple ongoing clinical trials (clinical trials: NCT02792465, NCT03568422 and NCT05251714) are now in place to validate the efficacy of small-molecule inhibitors of TTK as an antimitotic therapeutic target for several cancers [92]. Using a synthetic lethal RNA interference screen, the CTA ACRBP (acrosin binding protein) was identified in non-small cell lung (NSCLC) and ovarian cancers to be essential for bipolar spindle formation as a consequence of its regulation of NUMA accumulation [93,94]. A recent study also identified the CTA BAP31 (B-cell receptor-associated protein 31) as a CTA involved in the hyper-proliferation and metastatic phenotype of cervical cancer through its action on cell cycle progression and cytoskeletal assembly [95]. Other CTAs involved in proper cell abscission during cytokinesis (Centrosomal protein 55-CEP55 [83] and M-phase phosphorotein1-MPHOSPH1 [96]) and a group identified to positively regulate mitosis but the exact functions of which have yet to be elucidated (coiled-coiled domain-containing protein 110-CCDC110, FMR1 neighbour protein-FMR1NB, nuclear RNA export factor 2-NXF2, testis cancer centrosome-related protein-TCC52 and opa interacting protein 5-OIP5) [84,97,98] were also subsequently added to this growing list of cell-cycle regulatory CTAs.

In addition to their role in mitotic cell division, CTAs have also been implicated in meiosis. This is expected given that these proteins are expressed in germ cells where meiotic cell division exclusively occurs. CTAs such as SPO11(SPO11 initiator of meiotic double-strand breaks), TEX15 (testis expressed 15), SYCP1/3 (synaptonemal complex protein 1/3), HORMAD1/2 (HORMA domain containing 1/2) and, more recently, TEX12 (testis expressed 12) have all been shown to be essential for meiosis [99,100]. Expression of these genes in somatic or cancer cells has been correlated to either tumorigenesis, cancer progression or therapy resistance [101,102,103,104]. Of particular interest are SPO11, TEX15, SYCP1/3 and HORMAD1/2, all of which are involved in the DNA damage and repair response pathways during meiotic division. SPO11 is a component of the synaptonemal complex and its role involves the formation of double-stranded breaks to facilitate recombination events during meiosis [105]. TEX15 has been reported to function downstream of SPO11-mediated double-strand breaks (DSB) but upstream of RAD51- and DMC1-mediated (DNA meiotic recombinase1) DSB repair [106] and aberrant expression of TEX15 has been reported to be a risk factor for prostate cancer in the Han Chinese population [107]. SYCP1/3 and HORMAD1/2 directly affect the DNA damage response. SYCP1/3, a synaptonemal protein, was recently reported to interfere with BRAC2 (breast cancer gene 2) function and, thus, inactivates homologous recombination (HR), resulting in chromosomal instability [108]. HORMAD1/2, on the other hand, seems to have a role in both HR and non-homologous end joining (NHEJ), another DNA damage repair pathway. While in TNBCs, HORMAD1/2 was found to promote NHEJ, an error-prone repair pathway driving genomic instability in these cells [109]; it was observed to promote HR and support DNA repair and cell division in NSCLC [102]. Aberrantly expressed HORMAD1 was also shown to promote genomic instability in cancer cells by disrupting the nuclear localisation of the MCM8–MCM9 (mini-chromosome maintenance 8 and 9) complex, resulting in compromised DNA mismatched repair [110]. These studies suggest that, much like how TGFβ can be either oncogenic or tumour suppressive, the function of HORMAD1/2 in DNA repair is very much context-dependent and it can have opposing effects on DNA repair to influence malignant growth in different cell types. In a recent study, Sandhu and colleagues demonstrated that TEX12 misexpression in somatic cells could contribute to pathological amplification and dysfunction of centrosomes resulting in genomic instability and cancer progression [100].

In addition to promoting tumorigenesis, CTAs involved in DNA repair, or the DNA damage response (DDR), also promote the resistance of cancer cells to drugs or therapies targeting the DDR pathway when aberrantly expressed. Recent studies by our laboratory demonstrated that GAGE had the ability to confer resistance to radiation-induced cell death in cervical cancers. We found that GAGE expression levels were significantly higher in the tumours of patients resistant to radiation therapy. We further showed that GAGE, specifically the GAGE12 variant, augmented chromatin dynamics in cancer cells allowing for increased accessibility of DNA damage repair factors, which, upon induction of DNA damage, promoted more efficient DNA repair, protecting cells from DNA-damage-induced apoptosis [73]. Another study also demonstrated that TEX10 (testis expressed 10) promoted radioresistance in urinary bladder carcinoma by stabilising XRCC6 (x-ray repair cross complementing 6), a key factor required for efficient NHEJ upon induction of DNA damage by agents such as ionising radiation [111].

Abnormal chromosome segregation, aneuploidy, genomic instability and mutations are hallmarks of cancer. The combined effects of CTAs in mitosis, meiosis and DNA repair imply that their dysregulation in cancer cells may lead to increased chromosome missegregation or more error-prone DNA repair, driving genomic instability and carcinogenic mutations, although this has yet to be more rigorously proven. In addition, improved efficiency of DDR in cancer cells due to the aberrant expression of CTAs involved in DDR also contributes to therapy resistance, suggesting that targeting these CTAs may be considered in developing therapy resensitising strategies.

### 3.3. Role of CTAs in the Evasion of Apoptosis

From the first edition of the hallmarks of cancer, evasion of apoptosis has been recognised as an indispensable trait of cancer cells. Many studies have provided phenotypic evidence of CTA expression conferring some form of apoptosis resistance to a variety of cancer cells. To date, the role of how some of the more well-known CTAs aid in evading apoptosis in cancer cells are slowly beginning to emerge. Being the most studied CTA family, the function of MAGE proteins in multiple cellular pathways has been elucidated, including its role in regulating apoptosis. Apart from its earlier mentioned role in preventing p53 binding to its target promoters, MAGE was also found to be complex with other proteins affecting p53-mediated apoptosis. Specifically, recent studies indicate that MAGE family proteins can interact and regulate E3 ligase via its MHD domain, which binds to the RING domain of E3 ubiquitin ligases. MAGEC2 and MAGEA3/6 were identified to interact with the E3 ubiquitin ligase TRIM28/KAP1, leading to increased MDM2–p53 interaction, p53 degradation and loss of p53-mediated apoptosis [112,113,114]. In addition, MAGEA3 was reported to inhibit apoptosis via its action on p53-dependent BAX activation and stabilisation of surviving expression via both p53-dependent and independent mechanisms in multiple myeloma [115] and, more recently, in hepatocellular carcinoma [116]. Further, MAGEA4 was found to be associated with another E3 ligase, RAD18, promoting its stability and enhanced tolerance of cancer cells to DNA damage-induced apoptosis [117]. FATE1 (foetal and adult testis expressed 1) is another CTA implicated in apoptosis tolerance in tumour cells. The interaction of FATE1 with the E3 ligase RNF183 induced BIK (a BH3-only pro-apoptotic protein) degradation and subsequent resistance to apoptotic signals in cancer cells [69]. Recently, FATE1 was also found to confer resistance to calcium-induced programmed cell death via the uncoupling of endoplasmic reticulum (ER)–mitochondria activity. This was proposed as a possible mechanism of how FATE1 can contribute to chemotherapeutic resistance in cancer cells [118].

GAGE was first shown to have anti-apoptotic properties when it was demonstrated that overexpression of GAGE in HeLa cells conferred resistance to apoptosis induced by interferon-gamma (IFN-γ) or the death receptor Fas/CD95/APO-1. Although the activity of GAGE in the apoptotic pathway was mapped to be downstream of caspase-8 activation and downstream of PARP (poly (ADP-ribose) polymerase) cleavage, the exact molecular interactions and function of GAGE in the apoptotic pathway remain to be elucidated [119]. In 2009, Kular and colleagues attempted to define the mechanism of the earlier reported GAGE-mediated anti-apoptotic phenotype. In this study, the authors reported that GAGE downregulated interferon regulatory factor 1 (IRF1) and its target genes caspase-1 and caspase-7. They further showed that GAGE interacted with the multifunctional protein nucleophosmin (NPM)/B23 and this, combined with the downregulation of IRF1, provided resistance to IFN-γ-induced cell death on GAGE overexpressing cells [120]. Another CTA PAGE4 (prostate-associated gene 4), which is related to GAGE, was also shown to possess anti-apoptotic properties via its ability to regulate the cellular response to oxidative stress, with a report suggesting PAGE4 was able to suppress reactive oxygen species through p21 elevation [121]. More recently, it was demonstrated that PAGE4 promoted prostate cancer cell survival under oxidative stress by regulating the MAPK/JNK/ERK pathway [122].

The literature showing the phenotypic correlation between CTA expression and resistance to apoptosis is aplenty and beyond the scope of this review. However, the majority of the studies are still restricted to phenotypic presentations and correlations. Much more can be done to better elucidate the molecular underpinnings of how individual CTAs work in the cellular context to allow cancer cells to evade apoptosis, either during tumourigenesis or during the acquisition of therapy resistance. Filling this knowledge gap could improve treatment options, especially for therapy-resistant cancers.

### 3.4. CTAs and Metastasis: Epithelial-to-Mesenchymal Transition

A major obstacle in the clinical management of cancer is the ability of cancer cells to migrate out of primary tumour sites and plant themselves at distant sites of the human anatomy. Primary tumour cells must metastasise, proliferate and undergo angiogenesis at secondary sites to eventually form clinically relevant secondary tumours. Sperm cells are highly motile cells. To successfully fertilise the oocyte, these cells have to produce molecules that aid in the breaking down of components of the extracellular matrix (ECM) to enter the oocyte [123]. Many CTAs are involved in this process; thus, it is unsurprising that the reactivation of these CTAs in cancer cells has a role in promoting the metastatic phenotype.

Interestingly, CTAs are more commonly expressed in metastatic than primary tumours [124,125], underscoring their function in promoting the phenotypic presentations of metastasis. Epithelial-to-mesenchymal transition (EMT) is the process whereby cells lose their polarity and cell-to-cell adhesion, gaining motility, migratory and invasive properties. Various CTAs have been shown to enhance the EMT phenotype in cancer cells. SPANX A/C/D and CTAG2 were reported to work together to bring about the EMT phenotype in breast cancer cells, with CTAG2 interacting with Pericentrin at the centrosomes to influence directional migration. At the same time, SPANX A/C/D was observed to associate with Lamin A/C to reorganise the ECM [126]. In addition, other CTAs such as SSX (synovial sarcoma, X) and CAGE have been demonstrated to regulate key EMT-related genes such as *β-catenin* (*CTNNB1*) and *SNAIL* [127,128]. Of note, CAGE has also been implicated in the angiogenic process, a critical component of the multipathway process required for successfully forming secondary tumours at distant anatomical sites [129]. Recently, the CTAs MAGEC2, CT45A1 and PRAME were also reported to influence the EMT phenotype in cancer cells by modulating the expression of EMT genes [130,131,132,133]. PRAMEF2, a member of the PRAME family, was also shown to promote proliferation and the metastatic phenotype via its action on the HIPPO/YAP pathway in breast cancer [134]. A major pathway that promotes EMT is the WNT/β-catenin pathway and many CTAs have been demonstrated to affect this pathway directly or indirectly, contributing to the metastatic phenotype. Some recent examples include HORMAD1, recently shown to promote EMT in lung cancer by activating the WNT/β-catenin and AKT/GSK pathways [135] and the CTA PAGE4 via its inhibition of Tankyrase [136].

GAGE is highly expressed in the trophoblast and migrating primordial germ cells [137,138], suggesting their involvement in the migratory phenotype. GAGE expression has been reported to be correlated to the metastatic potential of breast and gastric cancer cells [139,140]. In gastric cancer, GAGE was hypothesised to have a role in regulating EMT genes, as the knockdown of *GAGE* resulted in the downregulation of several EMT genes. However, the exact mechanisms of how GAGE brings about this downregulation have not been studied and whether GAGE is directly involved in EMT gene regulation remains to be investigated [140]. Recently, GAGE was found to activate the p38δ/pMAPKAPK2/pHSP27 pathway in gastric cancer, contributing to increased proliferation and metastasis in gastric cancers [141].

The literature thus far suggests that CTAs may have an important role in the metastatic potential of cancer cells, in line with their proposed functions in gametogenesis, where their expressions usually correlate to a highly motile and invasive phenotype. Despite these phenotypic studies, the exact molecular involvement of CTAs in metastasis, EMT and angiogenesis is still an area that is murky and underexplored. Much more can be done to further our understanding of CTAs in metastasis. The molecular insights may prove vital for future applications of CTA-targeting therapeutics to improve disease outcomes.

### 3.5. Preferential Expression of CTAs in Cancer Stem Cells (CSCs)

CSCs are major contributors to tumorigenesis, therapy resistance and relapse in cancer patients. Two hypotheses exist as to the origins of the CSC population. One hypothesis suggests that CSCs arise from the normal stem cell population, while the other hints at differentiated cells regaining stem-like properties as the likely source of the CSC population. Evidence supporting both hypotheses has been demonstrated. Multiple studies have shown that CTAs are preferentially expressed in the stem-cell-like population in cancer tissue obtained from various cancer sites [142,143,144,145]. The preferential expression of highly immunogenic CTAs such as NY-ESO-1 in CSCs [144,146,147] is one of the reasons for the current interest in developing CTAs as prime targets for immunotherapy in cancer treatment. Apart from NY-ESO-1, a combination of other CTAs such as MAGE, GAGE, SPANX, XAGE, CT45A, SSX and more have been shown to be enriched in the side-populations/CSCs in a variety of cancers [142,143,144,148,149]. Several studies link the role of SSX to the biology of stem cells. SSX was reported to be recruited to the nuclear domains occupied by the Polycomb group (PcG) protein Bim-1 [150]. Bim-1 and the PcG complex are regulators of stem cell selfrenewal [151] and the colocalisation of SSX at PcG-occupied domains suggests a role for SSX in the selfrenewing properties of stem cells. Further, SSX has been reported to be expressed in mesenchymal stem cells (MSCs) and its expression confers migratory advantages to these cells. Knockdown of SSX was shown to induce mesenchymal to epithelial transition coupled with an increase in E-cadherin expression, suggesting its importance in maintaining the MSC phenotype [149]. The same study also observed that the expression of other CTAs, such as N-RAGE and NY-ESO-1, was downregulated when MSCs were induced to differentiate into adipocytes or osteocytes. Of note, there are currently no studies on the cellular functions of NY-ESO-1, which was originally reported to be localised in the cytoplasm [152]. In this study, NY-ESO-1 was observed to localise in nucleoli-like structures of the MSCs, suggesting possible novel functions and interactions of NY-ESO-1 in the nucleolus, which have yet to be identified. Although the functions of many CTAs in the maintenance of CSCs are still unexplored, some postulations can be made from known studies of CTA functions in germ cell development. For example, MAGEA [153] and MAGEB2 [154] were reported to protect germline cells from environmental stresses. Knockdown of *MAGEA* disrupts spermatogonial stem cell maintenance and impairs repopulation efficiency in vivo [153], while mouse ortholog of MAGE-B2 regulates stemness of testis spermatogonial stem cells and increases their ability for stress tolerance [154], suggesting upregulation of MAGE in CSCs may contribute to their maintenance and ability to travel to and repopulate at distal anatomical sites. More studies into how CTAs maintain primordial germ cell pluripotency and protect germ cells from environmental stresses could shed light on how CSCs utilise CTA reactivation to maintain their survival and repopulation advantage during cancer therapy.

### 3.6. CTAs in Cancer Cell Energetics and Autophagy

There are many shared characteristics between spermatogenesis and carcinogenesis regarding how they utilise energy metabolism to sustain cell proliferation [155]. Several CTAs are implicated in regulating energy production during spermatogenesis to meet the increased demand for motility [99]. Thus, CTA reactivation in cancers may have implications on cancer metabolism. A major organelle that drives cellular energetics is the mitochondria, the major “powerhouse” of the cell that provides the chemical energy needed to power essential biochemical processes for cell survival and proliferation. Several CTAs have been identified to be localised in the mitochondria (mitoCTAs). With in silico analysis suggesting that many CTAs contain putative mitochondria localisation signals in their amino acid sequences [155], it is reasonable to postulate that many of such CTAs could be involved in some capacity in mitochondria activity or biogenesis. Indeed, several recent studies have emerged to support this hypothesis. CTAs such as BORIS (shown to affect mitochondria fission and drive glycolysis in neuroblastoma cells) [156] and CT55 (which is involved in the maintenance of mitochondria DNA) [157] have been demonstrated to affect mitochondria integrity and quantity. While others, such as sperm-specific COX6B2 [158] and SEMG1/2 [159], have been described to affect mitochondria function directly through their actions on mitochondria complexes [158] or effects on mitochondria membrane function [159].

Otto Warburg first proposed in 1924 that cancer cells can rewire their metabolism towards the glycolytic pathway to promote the growth advantage of cancer cells even in a nutrient- and oxygen-deprived environment, a theory we now know as the Warburg effect [160]. Lactate dehydrogenases (LDH) play a critical role in regulating glycolysis and LDH-A and -C are two of the most commonly upregulated LDH in cancer cells. While LHD-A is usually expressed in somatic cells, LDH-C is expressed in the testis and cancer cells; thus, LDH-C was anointed as a CTA. LDH-C expression is upregulated in renal cancers [161] and associated with increased tumour invasion and metastasis in breast cancers [162]. MAGEAs are activated in many cancers where they partake in multiple cellular pathways, including those that promote fuel and metabolic switching. AMP-activated protein kinase (AMPK) is a master sensor and regulator of cellular energy levels. Recent studies have shown that MAGEA3/6 recruits the tumour-specific E3-ligase TRIM28 to AMPK, resulting in the degradation and loss of AMPK signalling. The loss of AMPK led to the downregulation of autophagy and upregulation of mTOR signalling, demonstrating how the reactivation of a CTA can alter cellular metabolism and act as an oncogenic driver [163].

Autophagy, an intracellular process involved in recycling and maintaining basal metabolite and intermediate biosynthetic levels in conditions of starvation, is an important mechanism for cellular adaptation in cancer cells. While it is now recognised that the reduction of autophagy could be vital in the transition of cells from “normal” to “cancer” [164], with the study on MAGE-A3/6 an example of this, it is also widely acknowledged that autophagy sustains metabolism in cancer cells under stress and the upregulation of autophagy in malignant cells provides some form of protection for cancer cells to therapy. The CTA FSIP1 (fibrous sheath interacting protein 1) is expressed in a majority of breast cancer tissues and is associated with poor prognosis [165,166]. In TNBC, it was demonstrated that FSIP1 interacts with ULK1 (Unc-51-like kinase1) to enhance AMPK-mediated autophagy, which promotes proliferation, invasion and resistance to chemotherapy [165]. Further, it was recently reported that the CTA TFDP3 belonging to the transcription factor DP (TFDP) family upregulates the expression of MAP1LC3, a key autophagic molecule, and increases the number of autophagosomes produced during chemotherapy in TNBC. The increase in autophagosome production was thought to promote the clearing of damaged organelles from cancer cells, increasing the tolerance of TNBC cells to chemotherapeutic drugs [167]. The CTA CAGE was also demonstrated to increase autophagic flux via its interaction with Beclin-1 and HER2, resulting in the resistance of PC9/HER2 cells to anticancer drugs [168]. Recent studies also suggest that CAGE mediated autophagy and drug resistance in gastric cancer via the CAGE–MiR-181b-5p–S1PR1 axis [169]. The sperm-associated antigen 6 (SPAG6) was also implicated in the regulation of autophagy via its action on the AMPK/mTOR/ULK1 signalling pathway [170].

These studies on CTAs in mitochondria dynamics and autophagy highlight the plasticity of the cancer cell genome and how reactivation of specific CTAs during specific phases of tumour progression can alter cellular metabolism and pathways. This genomic flexibility enables cancer cells to constantly adapt to extra- and intracellular insults to maintain their proliferative and survival advantage. Therefore, future studies on how and why CTAs are reactivated could provide valuable insights for the development of therapeutic strategies that target CTA re-expression to attenuate cancer cell adaptability to cytotoxic stimuli brought about by current anticancer drugs. In the long run, this will benefit our quest to combat treatment failure due to therapeutic resistance in the clinics.

### 3.7. CTAs and the Tumour Microenvironment

Cancer therapy was revolutionised with the discovery of cancer cell and host–immune system interactions. The tumour immune microenvironment is now an area of intense study with the rapid expansion of research focusing on immune-based therapy for cancer treatment. The specificity of expression and highly immunogenic nature of CTAs have positioned them as prime targets in immunotherapy, with several clinical trials [171,172] now underway. The vast number of CTAs are expected to provide a pool of antigenic peptides that bind to a wide range of HLA (human leukocytes antigens) molecules, which will induce humoral or cell-mediated immune responses in the human host to target tumour cells. NY-ESO-1 was discovered by SEREX in oesophageal cancers [35] and later identified to elicit both humoral and cellular responses in a high proportion of NY-ESO-1-expressing tumours in patients with different cancer types [173,174,175,176]. Several HLA class I- and II-restricted epitopes for NY-ESO-1 have been characterised. More epitopes are still emerging [177,178,179,180], making the NY-ESO-1 antigen a prototypic human cancer antigen in the development of antigen-specific human cancer vaccines [181], even though the biological functions of NY-ESO-1 remain unknown. However, some limitations to the efficacy of CTA-targeting immune-based therapy have emerged, especially with the discovery that many solid tumours appear nonpermissive to lymphocyte infiltration or are immunologically cold [182]. Interestingly, CTA expression has been demonstrated to correlate negatively to immune signature [145]. Recently, Shukla and colleagues elegantly demonstrated that a subcluster of MAGEA could predict resistance to CTLA-4 (cytotoxic T-lymphocyte associated protein 4) but not PD-1 (programmed death-1) blockade. Autophagy is thought to enhance the effects of CTLA-4-based immune therapy by optimising the release of immunostimulatory signals. MAGEA was previously shown to suppress autophagy [163]; thus, MAGEA expression was implicated in clinical resistance to CTLA-4 blockade, probably through its effects on autophagy regulation [183]. A subsequent study by Wang et al. further showed that MAGEA3 interacted with STAT1 (signal transducer and activator of transcription 1). These interactions resulted in the remodelling of the tumour microenvironment by affecting pathways involved in antigen presentation and immune cell infiltration [184].

Another extensively investigated target CTA for immunotherapeutic strategies is PRAME. Most studies on PRAME as an immune environment modulator implicate a negative correlation between the expressions of PRAME and antigen-presenting molecules as well as immune checkpoint molecules such as PD-1, LAG3 (lymphocyte-activation gene 3), PD-L1(programmed death-ligand 1), CD86 (cluster of differentiation 86), Gal-9 (galectin-9) and VISTA (V-domain Ig suppressor of T-cell activation) [185,186,187,188]. This implies that PRAME expression may be involved in immune escape mechanisms during carcinogenesis and may potentially indicate an immune cold tumour environment. Thus, modulation of PRAME expression is considered a viable strategy to improve the efficacy of immune checkpoint inhibitors in cancers. Interestingly, PRAME tumour expression was shown to suppress the expression and secretion of multiple proinflammatory cytokines and mediators of T-cell activation, differentiation and cytolysis during inflammation [185], further highlighting the role of PRAME in regulating immune activation in the tumour microenvironment (TME). Of note, a recent study by Takata et al. demonstrated that in patients with diffuse large B-cell lymphoma (DLBCL), *PRAME*-deleted tumours showed cytotoxic T-cell immune escape and were associated with cold tumour microenvironments with the enhancer of zeste homolog 2–activating (EZH2-activating) mutations suppressing PRAME expression. They further showed that PRAME interacted directly with EZH2 as a negative regulator affecting intracellular oncogenic pathways. EZH2 inhibition with EPZ-6438 abrogated these extrinsic and intrinsic effects, leading to PRAME expression and microenvironment restoration in vivo. Their model suggests dualistic functions of PRAME, including intracellular regulation (“intrinsic”) and TME modulation (“extrinsic”) as a consequence of frequently observed PRAME loss or reduced expression in DLBCL [189].

Given the complexity of the role of CTAs in genetic and signalling pathways, a shift in the thought process is warranted and CTAs may have a role in altering the immune landscape of cancer. Combining strategies targeting CTAs with current immune-therapy options may prove to be a viable approach in improving the response of tumours to currently available immunotherapeutic strategies.

Exosomes are small extracellular vesicles that act as important signalling molecules between tumour cells and other cells in the tumour microenvironment. Several studies are now emerging to suggest the presence of CTAs in exosomes. MAGEB4 was reported to show elevated expression in urinary exosomes from bladder cancer patients [190], while LDH-C4 was detected in exosomes isolated from serum obtained from breast cancer patients [191]. In addition, in TNBCs, the CTA SPANXB1 was exclusively found in extracellular vesicles and promoted the metastatic phenotype suggesting that CTAs secreted into the extracellular environment may remodel the tumour microenvironment to promote metastasis [192]. Although the exact biology of how these secreted CTAs in the tumour microenvironment affect tumour progression and therapy response remains to be elucidated; the fact that they can be readily detected in body fluids positions these CTAs as prime diagnostic targets for cancer detection using liquid biopsies.

## 4. Regulation of *CTAs*

*CTAs* are expressed in germ cells and trophoblasts and become silenced as they progress to differentiate into somatic cells. The coordinated silencing or expression of these *CTAs* during development suggests a common mechanism for regulating gene expression. There is now a large body of evidence implicating the epigenetic basis for the transcriptional regulation of *CTAs.* Global DNA hypomethylation occurs during gametogenesis when CTAs are most abundantly expressed [5]. Both global and promoter-specific, DNA hypomethylation has been associated with *CTA* expression in somatic and cancer cells [193,194]. Promoters of most *CTAs* contain regions enriched in CpG or CpG islands, suggesting DNA methylation as the primary mode of expression regulation for CTAs [195]. Weber and colleagues first demonstrated that *CTAs* were regulated by DNA methylation when they showed that the treatment of cells with 5-aza-2-deoxycytidine (decitabine) resulted in the upregulation of *MAGE1* expression [196] and other DNA methyltransferase inhibitors (DNMTi) such as zebularine were subsequently shown to activate *CTA* expression [197]. DNA hypomethylation-mediated *MAGEA11* activation was also shown to be involved in oncogenesis in prostate cancer [198]. Methylation patterns are inherited through cell generations and are maintained by DNA methyltransferases (DNMTs). Several studies have shown that manipulating DNMT levels in cells directly affected *CTA* expression patterns [199,200,201]. In addition, it was demonstrated that the downregulation of DNMT1 activated a methylated *MAGEA1* transgene in melanoma cells, strengthening the theory that DNA methylation is the primary mode of expression regulation for *CTAs* [202].

Apart from DNA methylation, histone modifications are also major mechanisms in the epigenetic regulation of *CTA* expression, with histone methyltransferases (HMTs) such as G9a and GLP demonstrated to regulate *CTAs* in embryonic stem cells and cancer cells [203,204]. Histone deacetylase inhibitors (HDACi) also seem to have a role in *CTA* regulation, with studies suggesting HDACi-mediated upregulation of *CTAs* and the potentiation of *CTA* upregulation with DNMTi–HDACi combinatory treatment [197,205]. The first direct evidence for the regulation of *CTA* expression via histone modifications was presented when mouse embryonic stem (ES) cells with a sustained knockout of *G9a*/*GLP* showed induced expression of *MAGEA* genes [206,207]. Interestingly, studies using inhibitors of histone modifiers revealed that the DNA methylation status seems to be a prerequisite for effective *CTA* upregulation by HMT activity or expression loss. Inhibition of GLP/G9a alone does not seem to induce *CTA* expression in cancer cells where DNA is more methylated. At the same time, robust upregulation was seen in ES cells where DNA tends to be hypomethylated [203,208,209]. A study later demonstrated that the enhancer of zeste homolog (EZH) 2, a histone methyltransferase and Polycomb group protein, was implicated in the repression of *GAGE* in breast cancer cell lines [210] and combined treatment with 3-deazaneplanocin A (DZNep), a potent inhibitor of EZH2, along with DNMTi and HDACi led to the robust induction of *GAGE* genes. This study further identified one breast cancer cell line with low endogenous DNA methylation levels but had low *GAGE* gene expression. In this particular cell type, it was discovered that EZH2-mediated H3K27 trimethylation influenced *GAGE* repression [210]. This observation suggests that while activation of *CTA* expression by histone modifications may be dependent on DNA methylation status, repression can be, in certain instances, exerted by histone modifiers even in the absence of DNA methylation changes. The latest studies have also shown that other histone modifiers, such as LSD1, can influence *GAGE* and other *CTA* gene expression in hepatocellular carcinoma through the growth-differentiation-factor-1 -dependent (GDF1) ALK/SMAD signalling cascade [211], further highlighting the importance of histone modifications in the epigenetic control of *CTA* expression in cancers.

Emerging studies are beginning to suggest that other non-epigenetic mechanisms of *CTA* regulation also exist in addition to epigenetic control. Transcription factors (TF) such as ETS TFs, SP1 and p53 have all been reported to affect the expression of *CTAs*. Two inverted ETS binding motifs were identified in the promoter of *MAGEA* [212,213]. While the exact ETS factor that binds to these motifs has yet to be determined, a study has suggested that ETS1 activates the expression of *MAGEA* genes with the prerequisite that the locus lacks DNA methylation and MBD1 binding [214], suggesting an interplay between DNA methylation and TF recruitment in the regulation of *CTA* expression. Many CTAs contain CpG-rich promoter regions, which makes them likely targets of Sp1 regulation. It was reported that Sp1 together with TFs CCCTC-binding factor (CTCF) and BORIS (which intriguingly is another CTA) work together to regulate the expression of the *NY-ESO-1* gene and mutation of Sp1 sequences diminished *NY-ESO-1* gene expression [215]. In addition, *BORIS* was reported to be regulated by p53 and CTCF in combination with CpG methylation [216]. Of note, while these studies suggest that TF-mediated *CTA* expression is an integral part of *CTA* gene regulation, TFs may need to work in concert with CpG methylation to exert their full effects.

**Figure 3:**
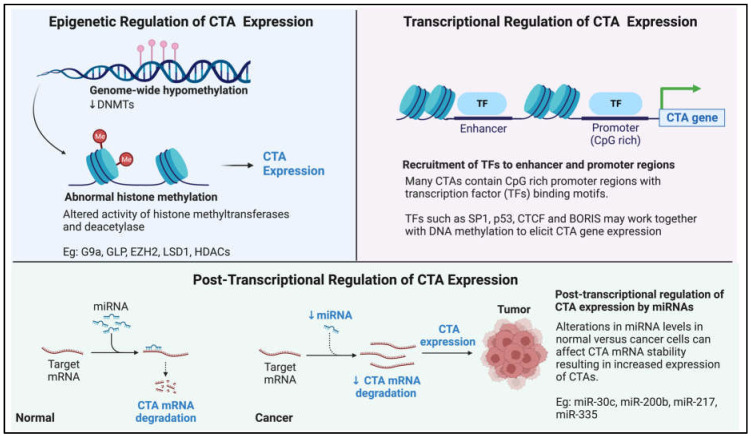
Pictorial summary of our current knowledge on how cancer-testis antigen (*CTA*) expression is regulated in the context of cancer.

Interestingly, recent studies have also implicated posttranscriptional regulation in the expression of CTAs. MicroRNAs (a class of non-coding RNAs), such as miR-30c, have recently been shown to regulate *GAGE* mRNA levels in gastric cancers [141]. The miRNAs miR-200b, miR-217 and miR-335 have also been demonstrated to have regulatory functions on *CAGE* expression [217]. In conclusion, there is still much to learn about how the expression of *CTAs* is regulated and how they become reactivated in tumorigenesis. Figure 3 provides a pictorial summary of the current known mechanism of *CTA* expression regulation.

## 5. CTAs as Therapeutic Targets: Perspectives and Future Implications

Traditional cancer therapy, including surgery, radiotherapy and chemotherapy, though relatively successful in the fight against cancer and prolonging the lifespan of patients, resistance, metastasis and relapse, still pose major obstacles to the clinical success of cancer treatment. Although recent advances in molecularly targeted therapeutics such as antibody-based targeting of the epidermal growth factor receptor (EGFR), human epidermal growth factor receptor 2 (Her2) and a cluster of differentiation 20 (CD20), which are currently used as standard targeted treatments in several cancers [218,219,220], have, to a certain extent, provided alternative options and improved treatment outcomes, the development of resistance after long-term use is still a major complication. Thus, there is still an urgent need for well-tolerated cancer treatment strategies that can induce durable clinical responses to be identified. As mentioned previously, the discovery and development of immune-based therapy was a breakthrough in cancer treatment, buoyed by the success of immune checkpoint inhibitors such as antibodies against programmed death-1 (PD-1) or its ligand, programmed death ligand-1 (PD-L1) and cytotoxic T-lymphocyte-associated antigen 4 (CTLA-4), which were shown to elicit a prolonged response and improve clinical outcome in several cancers [221].

**Figure 4:**
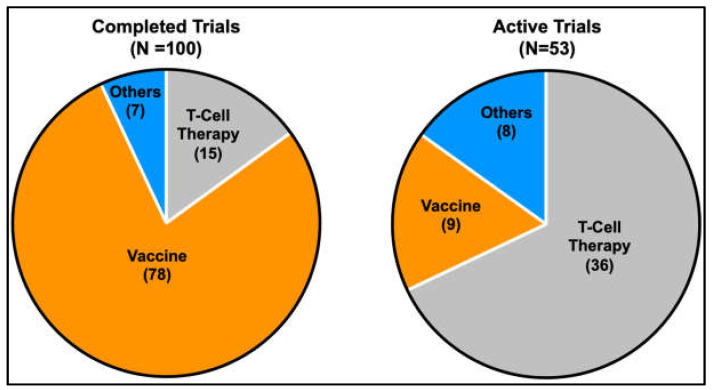
Pie charts indicating the proportion of current therapeutic strategies in completed and active clinical trials targeting CTAs. A gradual shift in the trend from more vaccine-based strategies to T-cell-based strategies is evident from the increase in the proportion of active trials that utilises T-cell-based strategies compared to earlier completed trials.(Information was obtained from https://clinicaltrials.gov, 6 March 2023).

Several clinical trials are underway to test the potential of vaccination-based immunotherapy using antigenic peptides against MAGE and NY-ESO-*1* to elicit cellular or humoral immune responses against various cancer types [171,172]. Recently, the use of ex vivo expanded autologous T-lymphocytes engineered to target intracellular antigens through T-cell receptors (TCRs) or cell surface antigens through chimeric antigen receptors (CARs) has also gained popularity [222,223]. The shift in trend towards T-cell-based therapy to target CTAs is evidenced by the increase in the proportion of active clinical trials that involve T-cell-based strategies. This contrasts with the higher proportion of CTA-targeted vaccine-based strategies in earlier completed clinical trials (Figure 4). The technology of targeting intracellular antigens through T-cell receptors (TCRs) is of particular interest when planning treatment strategies for targeting CTAs in cancer therapy, as CTAs are mainly intracellular antigens. Of note, tumour regression using genetically engineered T lymphocytes targeting NY-ESO-1 was successfully demonstrated in patients with metastatic synovial cell sarcoma and melanoma [224]. CTAs have an advantage over other intracellular antigens in adoptive T-cell therapy due to their restricted expression in tumour and immune-privileged germ cells, reducing potential complications from off-target toxicities. Amongst the various CTAs, PRAME, MAGEA3 and NY-ESO-1 have shown great potential as immunotherapeutic targets [225,226,227], with recent positive data coming from phase I clinical trials for the use of autologous T cells engineered to target the MAGEA4 showing promise in shrinking a wide range of solid tumours with a manageable toxicity profile [228]. Of note, the discovery of membrane-bound PRAME [229] sparked the development of the polyclonal antibody membrane-associated PRAME antibody 1 (MPA1) against the predicted extracellular PRA310-331 peptide. MPA1 has shown some promise in proof-of-concept studies targeting PRAME-expressing tumours with traditional antibody-based therapy [230]. Further, the identification of membrane-bound PRAME may also open avenues for targeting membrane-bound PRAME-expressing tumours using CAR T-cells.

Despite optimism about the potential therapeutic implications of these immune-based therapies, the success of such strategies may be hampered by factors such as loss or downregulation of HLA class I molecules, expression of immune checkpoint molecules, immune cell infiltration at tumour sites, low antigenicity of certain CTAs and heterogenicity of expression of CTAs in the bulk tumour. A possible way to circumvent some of the issues may be the use of epigenetic drugs as adjuvants to CTA-targeted immunotherapy [176]. The use of drugs such as Decitabine (DNA hypomethylating agent), Zebularine (DNMTi), HDAC inhibitors and DZNep (EZH2 inhibitor) may be considered as potential strategies to induce CTA expression in tumours to improve their susceptibility to CTA-targeting T-cell therapy. However, one must still be cautioned about the lack of specificity of these epigenetic drugs and their use could bring about unwanted off-target effects. Other strategies developed based on our understanding of the cellular functions of CTAs have also shown promise in cancer therapy. Examples would be using chemical compounds that block oncogenic MAGE ubiquitin ligase activity [231] and the use of TTK inhibitors to induce catastrophic mitotic events to bring about cell death in cancer cells expressing TTK [92]. Clinical trials have been initiated using the novel TTK inhibitor CFI-402257 [232] alone or in combination with paclitaxel in solid tumours (clinical trials: NCT02792465, NCT03568422 and NCT05251714). While we await the results of these trials, the proof-of-concept of such strategies in CTA-targeted therapy further drives home the need for a better understanding of the molecular basis of CTA functions to usher in new paradigms for the development of CTA-targeted therapeutics. Table 1 and Table 2 summarise the currently active clinical trials targeting CTAs.

## 6. Conclusions

Despite the improvements in sequencing technologies, artificial intelligence and the increasing amount of time and energy invested in functional studies of CTAs, several obstacles still lie in the way of our understanding of CTA biology. With most studies thus far focused on expression patterns in small sample sizes, the lack of large-scale transcriptomics data from normal tissues and insufficient numbers of high-quality cancer samples has made it challenging to evaluate *CTA* genes systematically and comprehensively [39]. Further, while hundreds of genes are listed in databases, much of the function of these genes in normal development and gametogenesis remains elusive, with many studies showing correlations to the cancer phenotype but lacking in mechanistic understanding. Hampered by the lack of murine orthologs of many CTAs, such as GAGE, finding the most relevant physiological models for mechanistic and functional studies remain a major roadblock that needs to be circumvented. In addition, how these genes are reactivated in cancers and if these genes indeed support tumorigenesis, disease progression and therapy resistance remain to be thoroughly explored. Clinically, while CTA-targeted therapy has been an area of intense interest, of the potential 200 CTA targets, only a handful have gone on to be tested in clinical trials for their efficacy. More can definitely be done to improve our understanding of CTAs. How we can utilise knowledge gained in future studies to develop CTA-targeted anticancer strategies will be a crucial area for further exploration.

## Figures and Tables

**Table 1:** List of currently recruiting clinical trials targeting CTAs.

Therapy	NCT Number	Title	Cancer Type	Target CTA	Sponsor	Location
T-Cell	NCT05430555	A Phase 1/2, First-in-Human, Open-Label, Two-Part Clinical Trial of TK-8001 in Patients With HLA-A*02:01 Genotype and Advanced-Stage/Metastatic MAGE-A1+ Solid Tumors	Advanced Solid tumours	MAGEA1	T-knife GmbH	Berlin, Germany
	NCT03240861	Genetically Engineered PBMC and PBSC Expressing NY-ESO-1 TCR After a Myeloablative Conditioning Regimen to Treat Patients With Advanced Cancer	Malignant Neoplasm/ Sarcoma	NY-ESO-1	Jonsson Comprehensive Cancer Center	CA, USA
	NCT04752358	ADP-A2M4CD8 in HLA-A2+ Subjects With MAGE-A4 Positive Esophageal or Esophagogastric Junction Cancers (SURPASS-2)	Esophageal Cancer/ Esophagogastric Junction Cancer	MAGEA4	Adaptimmune	UK
	NCT04729543	MAGE-C2 TCR T Cell Trial to Treat Melanoma and Head and Neck Cancer	Melanoma/ Head and Neck Cancer	MAGEC2	Erasmus Medical Center	Rotterdam, Netherlands
	NCT05296564	Anti-NY-ESO-1 TCR-Gene Engineered Lymphocytes Given by Infusion to Patients With NY-ESO-1 -Expressing Metastatic Cancers	Metastaic solid tumours	NY-ESO-1	Hadassah Medical Organization	Jerusalem, Israel
	NCT04044859	ADP-A2M4CD8 as Monotherapy or in Combination With Either Nivolumab or Pembrolizumab in HLA-A2+ Subjects With MAGE-A4 Positive Tumors (SURPASS)	Multiple Solid Tumors	MAGEA4	Adaptimmune	UK
	NCT03652545	Multi-antigen T Cell Infusion Against Neuro-oncologic Disease	Brain Tumor	WT1, PRAME	Catherine Bollard	WA, USA
	NCT04884282	Efficacy of Tedopi Plus Docetaxel or Tedopi Plus Nivolumab as Second-line Therapy in Metastatic Non-small-cell Lung Cancer Progressing After First-line Chemo-immunotherapy (Combi-TED)	Metastatic Non-Small Cell Lung Cancer	MAGE2/3	Fondazione Ricerca Traslazionale	Roma, Italy
	NCT05134740	(TAA)-Specific Cytotoxic T-Lymphocytes to Pediatric Patients With Lymphomas (pediTACTAL).	Hodgkins Lymphoma, Non Hodgkins Lymphoma	NY-ESO-1, MAGEA4, PRAME, SSX.	Baylor College of Medicine	TX, USA
	NCT05620693	Study of NY-ESO-1 TCR-T in Advanced Soft Tissue Sarcoma	Advanced Soft-tissue Sarcoma	NY-ESO-1	Shenzhen University General Hospital	Shenzhen, China
	NCT03462316	NY-ESO-1-specific T Cell Receptor (TCR) T Cell in Sarcoma	Bone Sarcoma, Soft Tissue Sarcoma	NY-ESO-1	Sun Yat-sen University	Guangzhou, China
	NCT05549921	Phase II Study of TAEST16001 in Soft Tissue Sarcoma	Soft Tissue Sarcoma	NY-ESO-1	Sun Yat-sen University	Shenzhen, China
	NCT02494167	Administration of Donor Multi TAA-Specific T Cells for AML or MDS (ADSPAM)	Acute Myeloid Leukemia, Myelodysplastic Syndrome	NY-ESO-1	Baylor College of Medicine	TX, USA
	NCT04044768	Spearhead 1 Study in Subjects With Advanced Synovial Sarcoma or Myxoid/Round Cell Liposarcoma	Synovial Sarcoma, Myxoid Liposarcoma	MAGEA4	Adaptimmune	UK
	NCT01946373	T Cell Transfer With or Without Dendritic Cell Vaccination in Patients With Melanoma	Melanoma	NY-ESO-1	Karolinska University Hospital	Slona, Sweden
	NCT03686124	ACTengine^®^ IMA203/IMA203CD8 as Monotherapy or in Combination With Nivolumab in Recurrent and/or Refractory Solid Tumors	Solid Tumor, Recurrent Solid Tumors, Refractory Solid Tumors	PRAME	Immatics US, Inc.	TX, USA
	NCT05035407	T Cell Receptor Gene Therapy Targeting KK-LC-1 for Gastric, Breast, Cervical, Lung and Other KK-LC-1 Positive Epithelial Cancers	Gastric, Breast, Cervical, Lung and Other KK-LC-1 Positive Epithelial Cancers	KK-LC-1	National Cancer Institute (NCI)	MD, USA
Vaccine	NCT04908111	A Trial of ChAdOx1 and MVA Vaccines Against MAGE-A3 and NY-ESO-1	Non-small Cell Lung Cancer	MAGEA3, NY-ESO-1	Cancer Research UK	London, UK
	NCT04739527	Phase 1 Study to Evaluate the Safety, Feasibility and Immunogenicity of an Allogeneic, Cell-based Vaccine (DCP-001) in High Grade Serous Ovarian Cancer Patients After Primary Treatment	Ovarian Cancer	WT1, PRAME	University Medical Center Groningen	Groningen, Netherland
	NCT03970746	Safety, Immunogenicity and Preliminary Clinical Activity Study of PDC*lung01 Cancer Vaccine in NSCLC	Non Small Cell Lung Cancer	NY-ESO-1, MAGEA3, MAGEA4, Multi-MAGE	PDC*line Pharma SAS	Brussels, Belgium
	NCT04751786	Dose Escalation Study of Immunomodulatory Nanoparticles	Advanced Solid Tumor	NY-ESO-1	Radboud University Medical Center	Nijmegen, Netherlands
Others	NCT03973333	Safety and Efficacy of IMC-C103C as Monotherapy and in Combination With Atezolizumab	Select Advanced Solid Tumors	MAGEA4	Immunocore Ltd.	Oxfordshire, UK
	NCT04262466	Safety and Efficacy of IMC-F106C as a Single Agent and in Combination With Checkpoint Inhibitors	Select Advanced Solid Tumors	PRAME	Immunocore Ltd.	Oxfordshire, UK
	NCT05251714	CFI-402257, a Potent and Selective TTK Inhibitor, in Solid Tumors and With Fulvestrant in Breast Cancer	Advanced Solid Tumors, Breast Cancer	TTK	Treadwell Therapeutics, Inc.	NY, USA

Information was obtained from https://clinicaltrials.gov (accessed on 6 March 2023).

**Table 2:** List of currently active but not recruiting clinical trials targeting CTAs.

Therapy	NCT Number	Title	Cancer Type	Target CTA	Sponsor	Location
T-Cell	NCT03139370	Safety and Efficacy of MAGE-A3/A6 T Cell Receptor Engineered T Cells (KITE-718) in HLA-DPB1*04:01 Positive Adults With Advanced Cancers	Multiple Solid Tumors	MAGEA3/A6	Kite, A Gilead Company	CA, USA
	NCT03132922	MAGE-A4ᶜ¹º³²T for Multi-Tumor	Solid tumours	MAGEA4	Adaptimmune	UK
	NCT02869217	Study of TBI-1301 (NY-ESO-1 Specific TCR Gene Transduced Autologous T Lymphocytes) in Patients With Solid Tumors	NY-ESO-1 Expressing Solid Tumors	NY-ESO-1	University Health Network, Toronto	Toronto, Canada
	NCT03093350	TACTIC - TAA Specific Cytotoxic T Lymphocytes in Patients With Breast Cancer	Breast Cancer	NY-ESO-1, MAGEA4, PRAME, SSX2	Baylor College of Medicine	TX, USA
	NCT02650986	Gene-Modified T Cells With or Without Decitabine in Treating Patients With Advanced Malignancies Expressing NY-ESO-1	Advanced Solid tumours	NY-ESO-1	Roswell Park Cancer Institute	NY, USA
	NCT03192462	TAA Specific Cytotoxic T Lymphocytes in Patients With Pancreatic Cancer	Pancreatic Cancer	NY-ESO-1, MAGEA4, PRAME, SSX2	Baylor College of Medicine	TX, USA
	NCT03691376	Genetically Engineered Cells (NY-ESO-1 TCR Engineered T Cells and HSCs) After Melphalan Conditioning Regimen in Treating Patients With Recurrent or Refractory Ovarian, Fallopian Tube, or Primary Peritoneal Cancer	Recurrent or Refractory Ovarian, Fallopian Tube, or Primary Peritoneal Cancer	NY-ESO-1	Roswell Park Cancer Institute	NY, USA
	NCT04526509	Master Protocol to Assess Safety and Dose of First Time in Human Next Generation Engineered T Cells in NY-ESO-1 and/or LAGE-1a Positive Advanced Solid Tumors	Neoplasms	NY-ESO-1and LAGE-1a	GlaxoSmithKline	London, UK
	NCT03967223	Master Protocol to Assess the Safety and Antitumor Activity of Genetically Engineered T Cells in NY-ESO-1 and/or LAGE-1a Positive Solid Tumors	Neoplasms	NY-ESO-1	GlaxoSmithKline	London, UK
	NCT03017131	Genetically Modified T Cells and Decitabine in Treating Patients With Recurrent or Refractory Ovarian, Primary Peritoneal, or Fallopian Tube Cancer	Recurrent or Refractory Ovarian, Primary Peritoneal, or Fallopian Tube Cancer	NY-ESO-1	Roswell Park Cancer Institute	NY, USA
	NCT04318964	TAEST16001 in the Treatment of Soft Tissue Sarcoma	Soft Tissue Sarcoma	NY-ESO-1, LAGE-1	Sun Yat-sen University	Guangzhou, China
	NCT03247309	TCR-engineered T Cells in Solid Tumors (ACTengine IMA201-101)	Solid Tumor, Recurrent Solid Tumors, Refractory Solid Tumors	MAGEA4/A8	Immatics US, Inc.	TX, USA
	NCT01333046	Administration of TAA-Specific CTLs; Hodgkin or Non-Hodgkin Lymphoma; TACTAL	Hodgkin Lymphoma, Non-Hodgkin Lymphom, |Hodgkin Disease	NY-ESO-1, MAGEA4, PRAME, SSX.	Baylor College of Medicine	TX, USA
	NCT03441100	TCR-engineered T Cells in Solid Tumors: IMA202-101	Solid Tumor, Recurrent Solid Tumors, Refractory Solid Tumors	MAGEA1	Immatics US, Inc.	TX, USA
	NCT03450122	Modified T Cells, Chemotherapy, and Aldesleukin With or Without LV305 and CMB305 in Treating Participants With Advanced or Recurrent Sarcoma	Advanced or Recurrent Sarcoma	NY-ESO-1	M.D. Anderson Cancer Center	TX, USA
	NCT04679194	Study of Mana 312 (Multi Tumor-Associated Antigen T Cells) in Adults With AML/MDS After HSCT	AML/MDS	WT1, PRAME	Mana Therapeutics	VA, USA
	NCT03250325	Study of TBI-1301 (NY-ESO-1 T Cell Receptor Gene Transduced Autologous T Lymphocytes) in Patients With Synovial Sarcoma	Synovial Sarcoma	NY-ESO-1	Takara Bio Inc.	CA, USA
	NCT02475707	Administration of Donor MultiTAA-Specific T Cells for ALL (STELLA)	Leukemia, Lymphoblastic (Acute)	WT1, NY-ESO-1, PRAME	Baylor College of Medicine	TX, USA
Vaccine	NCT02737787	A Phase I Study of WT1 or NY-ESO-1 Vaccine and Nivolumab For Recurrent Ovarian Cancer	Ovarian, Fallopian Tube, Primary Peritoneal Cancer, Recurrent Ovarian Cancer	WT1, NY-ESO-1	Memorial Sloan Kettering Cancer Center	NY, USA
	NCT01697527	Gene and Vaccine Therapy in Treating Patients With Advanced Malignancies	Malignant Neoplasm	NY-ESO-1	Jonsson Comprehensive Cancer Center	CA, USA
	NCT02410733	Evaluation of the Safety and Tolerability of i.v. Administration of a Cancer Vaccine in Patients With Advanced Melanoma (Lipo-MERIT)	Melanoma	MAGEA3, NY-ESO-1	BioNTech SE	Mainz, Germany
	NCT03206047	Atezolizumab, Guadecitabine, and CDX-1401 Vaccine in Treating Patients With Recurrent Ovarian, Fallopian Tube, or Primary Peritoneal Cancer	Platinum-Resistant or Recurrent Fallopian Tube, Ovarian, Primary Peritoneal Carcinoma	NY-ESO-1	National Cancer Institute (NCI)	MD, USA
	NCT01176474	Vaccine Combining Multiple Class I Peptides and Montanide ISA 51VG With Escalating Doses of Anti-PD-1 Antibody Nivolumab or Ipilimumab With Nivolumab For Patients With Resected Stages IIIC/IV Melanoma	Melanoma (Skin)	NY-ESO-1	H. Lee Moffitt Cancer Center and Research Institute	FL, USA
Others	NCT02285816	MG1 Maraba/MAGE-A3, With and Without Adenovirus Vaccine With Transgenic MAGE-A3 Insertion in Incurable MAGE-A3-Expressing Solid Tumours	Advanced or Metastatic Solid Tumours	MAGEA3	Canadian Cancer Trials Group	ON, Canada
	NCT04939701	Study of ASP0739 Alone and With Pembrolizumab in Advanced Solid Tumors With NY-ESO-1 Expression Participants	Ovarian Cancer, Non Small Cell Lung Cancer, Esophageal Squamous-Cell Carcinomas, Solid Tumours	NY-ESO-1	Astellas Pharma Global Development, Inc.	IL, USA
	NCT02792465	A Study of Investigational Drug CFI-402257 in Patients With Advanced Solid Tumors	Breast Cancer	TTK	University Health Network, Toronto	Toronto, Canada
	NCT03568422	CFI-402257 in Combination With Paclitaxel in Patients With Advanced/Metastatic HER2-Negative Breast Cancer	Breast Cancer	TTK	Canadian Cancer Trials Group	ON, Canada
	NCT05129280	A Study to Evaluate Safety, Pharmacokinetics, and Preliminary Anti-tumor Activity of RO7444973 in Participants With Unresectable and/or Metastatic MAGE-A4-positive Solid Tumors	Solid tumours	MAGEA4	Hoffmann-La Roche	Switzerland

Information was obtained from https://clinicaltrials.gov (accessed on 6 March 2023).

## Data Availability

Not applicable.
